# Intrahepatic cholangiocarcinoma in a non-cirrhotic liver in a patient with homozygous ZZ alpha-1 antitrypsin deficiency

**DOI:** 10.1136/bcr-2020-240077

**Published:** 2021-03-03

**Authors:** Nienke E Vuurberg, Anne Loes Van den Boom, Marius C Van den Heuvel, Joost M Klaase

**Affiliations:** 1 Department of Surgery, Division of Hepato-Pancreato-Biliary Surgery and Liver Transplantation, University Medical Centre Groningen, Groningen, The Netherlands; 2 Department of Pathology, University Medical Centre Groningen, Groningen, The Netherlands

**Keywords:** surgery, surgical oncology, liver disease, hepatic cancer

## Abstract

Alpha-1 antitrypsin (AAT) deficiency, which is an under-recognised metabolic genetic disorder, is known to cause severe lung disease and liver cirrhosis in about 10%–15% of cases. Patients with AAT deficiency are at a higher risk for developing hepatocellular carcinoma, both in cirrhotic and in non-cirrhotic livers. In this case report, a 48-year-old woman with homozygous ZZ AAT deficiency presented with abdominal pain, and by imaging, an abnormal area in the liver was found. The initial differential diagnosis consisted of benign abnormalities but a malignancy could not be ruled out. Finally, this abnormality turned out to be an intrahepatic cholangiocarcinoma (iCCA) in a non-cirrhotic liver. Since this type of tumour has been very infrequently described to be associated with AAT deficiency, the question remains whether alpha-1 trypsin accumulation in the hepatocytes was responsible for the development of iCCA. However, other associated factors for developing an iCCA were ruled out.

## Background

Alpha-1 antitrypsin (AAT) deficiency is an under-recognised metabolic autosomal recessive genetic disorder that affects the lung, liver and skin.^1^ Multiple gene expressions are known in AAT deficiency, whereas mutations in the Z and S alleles are the most common.^2–4^ Patients with AAT deficiency with low AAT levels, which is most common in patients with the homozygous ZZ phenotype, are at risk for developing symptoms like shortness of breath, abdominal pain or jaundice.^2 3^ The effect of heterozygous mutations is controversial but several studies show an increased risk for liver cancer in these patients (see table 1).^1–4^ AAT is a serine protease inhibitor that is produced in hepatocytes and secreted into the serum. A deficiency of AAT may cause loss of elastase function in the lungs leading to emphysema/chronic obstructive pulmonary disease (COPD). In the liver, the relation between AAT deficiency and the pathogenesis of (progressive) liver disease is unclear. The deficiency leads to a defective way of protein misfolding and erroneous export from the hepatocytes, the major cellular source of circulating AAT. This causes retention and polymers that accumulate in the endoplasmic reticulum of the hepatocytes rather than being efficiently secreted.^1 2^ Homozygous and heterozygous children and adults may develop liver disease and hepatocellular carcinoma (HCC), which appears not to be related to the existence of liver cirrhosis.^1–6^ On the other hand, a second mutational hit could be necessary in the progression to carcinoma.^7^

**Table 1:** An overview of the articles described in the discussion

Article	Described relation of AAT and cholangiocarcinoma	Pre-existing liver tissue	Phenotype	Number of patients
Parham *et al*^8^	Two siblings with AAT deficiency showed histological features of cholangiocarcinoma.	One patient with early cirrhosis, the other had no cirrhosis	Homozygous PiZZ	2
Zhou *et al*^3^	Seven CCA and four CHCC out of 317 consecutive primary liver carcinomas showed AAT deficiency.	No cirrhosis	Heterozygous PiZ	7
Angkathunyakul *et al*^6^	A relation between AAT-deficient patients and biliary lesions was shown but not sufficient to warrant a diagnosis of cholangiocarcinoma.	No cirrhosis	Homozygous PiZZ	1
Mihalache *et al*^1^	17 of 182 patients with cholangiocarcinoma had AAT deficiency vs 12 of 290 patients without cholangiocarcinoma.	Unknown	Heterozygous PiZ	17

AAT, alpha-1 antitrypsin; CCA, cholangiocarcinoma; CHCC, combined hepatocholangiocarcinoma.

Cholangiocarcinoma (CCA) is rarely described in association with AAT. Intrahepatic CCA (iCCA) is a devastating cancer arising from the epithelial lining of intrahepatic bile ducts with rising incidence rates worldwide.^1 8^ Most frequent risk factors for (i)CCA are biliary diseases like primary sclerosing cholangitis, hepatobilary flukes, hepatolithiasis, bile duct cystic disorders, cirrhosis, hepatitis B and C, and chronic biliary inflammation.^9^ In addition, several environmental and toxic causes such as alcohol and smoking are known risk factors.^10^ The clinical course of iCCA may present differently from extrahepatic CCA. The latter causes symptoms related to biliary obstruction including jaundice, pruritus, clay-coloured stools and dark urine, whereas iCCA is less likely to cause jaundice. Instead, they usually have a history of dull right upper quadrant pain, weight loss and an elevated alkaline phosphatase.^6 7 11^ Currently, the prognosis of CCA is poor due to diagnosing iCCA in advanced stage of the disease and limited treatment methods. Among patients who undergo potentially curative resection for CCA, long-term outcomes vary according to location and stage of the primary lesion; therefore, early detection is important.^12^

## Case presentation

Our patient presented with episodes of abdominal pain and different defecation patterns. The 48-year-old female patient had a medical history of laparoscopic cholecystectomy. Furthermore, she was screened for additional risk factors for iCCA but, except for the known AAT deficiency, nothing was found. She and her brother were known to have AAT deficiency, as genetics showed a homozygous ZZ allele. Her brother underwent a lung transplantation; she never had any physical complaints related to this disease with good lung function. Her diagnosis of AAT deficiency was made after consulting genetics.

## Investigations

CT and MRI of the abdomen showed an abnormal area in segment 5 of the liver with capsular withdrawal, focal bile duct dilatation and late (fibrotic) colouring. These findings led to a differential diagnosis of a benign aetiology (inflammatory pseudomass, focal fibrosis after, eg, a vascular incident or cholangitis); however, an iCCA could not be ruled out. These image modalities and laboratory findings did not show any sign of cirrhosis. A fibroscan was made to indicate if the remnant liver tissue consisted of normal tissue. Laboratory results were likewise normal and tumour markers were normal (alfa-1-fetopoprotein 1.4 U/mL, carcinoembryonic antigen (CEA) 4.8 mg/mL and cancer antigen 19.9 11 kU/L). No Endoscopic retrograde cholangiopancreatography was performed since this would not yield additional information and drainage was not needed.

## Treatment

**Figure 1:**
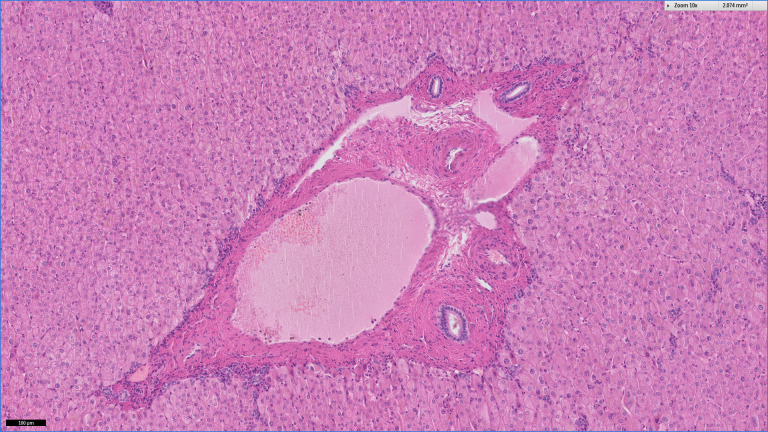
H&E image of normal portal tract.

**Figure 2:**
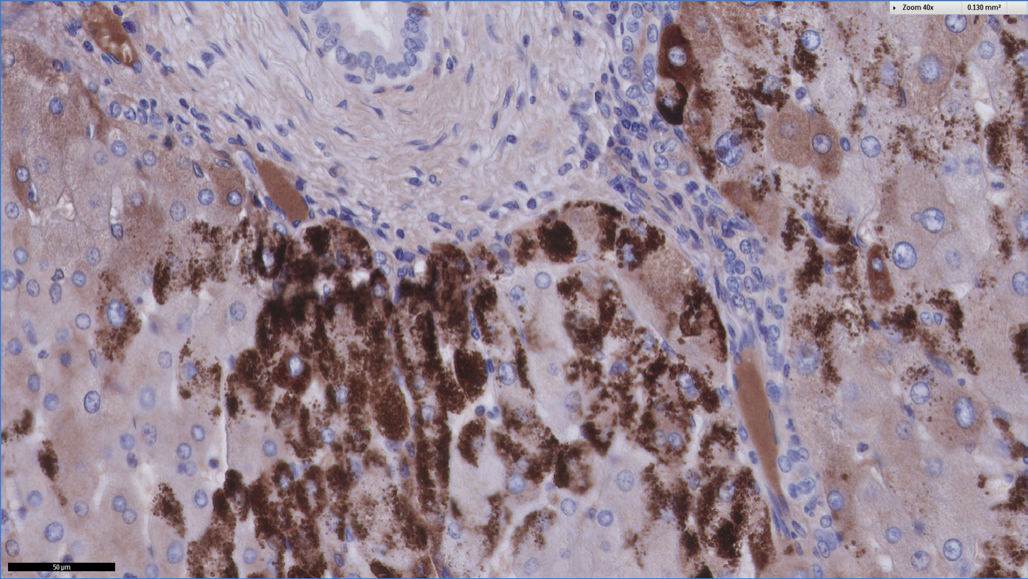
Immunohistochemistry with alpha-1 antitrypsin, positive globules in periportal hepatocytes.

**Figure 3:**
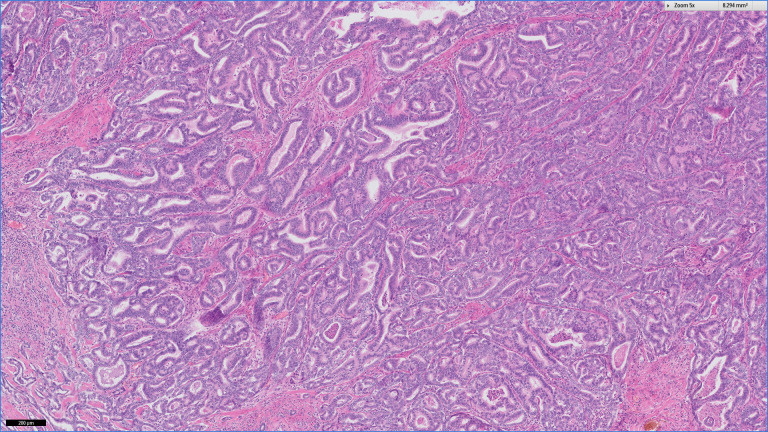
Overview of the cholangiocarcinoma. The tumour is composed of ductular structures with variation in size and form.

After multidisciplinary considerations, it was decided to perform a right hemihepatectomy. Pathology examination confirmed the diagnosis of a well-differentiated iCCA, with a tumour mass of 4.3 cm (figures 1–3). Pre-existent liver tissue showed accumulation of AAT in periportal hepatocytes. There were no signs of fibrosis or cirrhosis.

## Outcome and follow-up

The postoperative course was complicated by an unplanned readmission because of chylous leakage treated with percutaneous drainage. Follow-up consists of MRI every 4 months. No adjuvant treatment was given.

## Discussion

There is a lack of information about (i)CCA in patients with AAT, only case reports are published. Parham *et al*^8^ described two siblings with homozygous AAT and alcohol abuse, developing CCA. Biopsy of the liver lesion of the man (at the age of 38 years) showed a dense fibrous tissue containing malignant cells, most probably CCA rather than HCC. Autopsy of the female patient (28 years old) showed macroscopic and histological features of metastatic CCA. Whereas in the male patient signs of early cirrhosis were seen, the female patient showed no abnormalities in the pre-existent liver tissue. Zhou *et al*^3^ analysed a total of 3010 liver specimens. These specimens consisted of 317 primary liver carcinomas (225 HCC, 20 combined hepatocholangiocarcinoma (CHCC), 72 CCA) with normal liver tissue as well, and 1663 liver biopsies together with 1030 autopsies as control series. In the resection specimens of primary liver carcinomas, significantly more AAT deposits in the liver epithelia (5.99%) were found compared with the control series (biopsies 3.43% p=0.03; autopsies 1.84% p<0.001). The percentage of CCA (n=7) or CHCC (n=4) was significantly higher in liver resections with AAT (n=19) compared with resections without AAT (p=0.004). All seven CCA developed in a non-cirrhotic liver of patients who were heterozygous for AAT deficiency. This demonstrates that malignancies can occur in a non-cirrhotic liver of patients with AAT deficiency. Angkathunyakul *et al*^6^ suggested a relation between AAT deficiency and biliary lesions. They investigated 11 biliary lesions in five patients with AAT deficiency and found frequent BRAF V600E mutations, supporting their potential to progress to biliary malignancy. Mihalache *et al*^1^ showed 17 patients diagnosed with CCA and AAT deficiency. No significant relationship between AAT deficiency and CCA could be demonstrated in this study.

To conclude, only 26 cases of CCA in patients with AAT deficiency are described so far. Reviewing the literature suggests that the appearance of CCA could be linked to AAT deficiency; however, the evidence is not evidential. An overview of the described articles is displayed in table 1.

### Current guidelines of surveillance in patients with AAT deficiency

AAT deficiency is associated with a number of conditions; therefore, the clinical evaluation of a patient with AAT deficiency should pay special attention to the early detection and follow-up of these associated conditions. After diagnosis, patients should first be assessed for liver and pulmonary function.^13 14^ Sandhaus *et al* recommend individuals with AAT deficiency to be monitored for liver disease at annual intervals (or more frequently as indicated by clinical circumstances), with physical examination including a focused examination for signs of liver disease, laboratory monitoring and liver ultrasound.^13^ Guidelines call for regular screening that includes ultrasound examination of the liver, in AAT patients with cirrhosis, every 6 months.^14^

**Learning points**

- Consider liver malignancy in patients with alpha-1 antitrypsin (AAT) deficiency, even without cirrhosis.
- Beside hepatocellular carcinoma, which is the most common liver disorder known in AAT deficiency, cholangiocarcinoma should be included in the differential diagnosis.
